# The effects of individual and community-level factors on community-based health insurance enrollment of households in Ethiopia

**DOI:** 10.1371/journal.pone.0275896

**Published:** 2022-10-10

**Authors:** Yikeber Abebaw Moyehodie, Solomon Sisay Mulugeta, Seyifemickael Amare Yilema

**Affiliations:** Department of Statistics, Debre Tabor University, Debre Tabor, Ethiopia; University of Bern, SWITZERLAND

## Abstract

**Introduction:**

Community-based health insurance (CBHI) is a type of volunteer health insurance that has been adopted all over the world in which people of the community pool funds to protect themselves from the high costs of seeking medical care and treatment for the disease. In Ethiopia, healthcare services are underutilized due to a lack of resources in the healthcare system. The study aims to identify the individual and community level factors associated with community-based health insurance enrollment of households in Ethiopia.

**Methods:**

Data from the Ethiopian mini demographic and health survey 2019 were used to identify factors associated with community-based health insurance enrollment of households in Ethiopia. Multilevel logistic regression analysis was used on a nationally representative sample of 8,663 households nested within 305 communities, considering the data’s layered structure. We used a p-value<0.05 with a 95% confidence interval for the results.

**Result:**

The prevalence of community-based health insurance enrollment in Ethiopia was 20.2%. The enrollment rate of households in the scheme was high in both Amhara (57.9), and Tigray (57.9%) regions and low (3.0%) in the Afar region. At the individual level; the age of household heads, number of children 5 and under, number of household members, has land for agriculture, has a mobile telephone, receiving cash of food from the safety Net Program, Owning livestock, and herds of farm animals, wealth index, and at the community level; the region had a significant association with community-based health insurance enrollment.

**Conclusion:**

Both individual and community-level characteristics were significant predictors of community-based health insurance enrollment in households. Furthermore, the ministry of health, health bureaus, and other concerning bodies prioritize clusters with low health insurance coverage to strengthen health system financing and intervene in factors that negatively affect the CBHI enrollment of households.

## Introduction

Community-based health insurance (CBHI) is a type of volunteer health insurance that has been adopted all over the world in which people of the community pool funds to protect themselves from the high costs of seeking medical care and treatment for the disease. It improves healthcare utilization and protects households from out-of-pocket costs [1–3]. For a long time, Universal Health Coverage (UHC) has been a global concern. The World Health Organization (WHO) and other international organizations have suggested achieving UHC, mainly through health insurance. The WHO defines UHC as ensuring that all people have access to essential promotive, preventive, curative, and rehabilitative health services of adequate quality to be effective and ensuring that their usage does not place people in financial difficulty. However, most healthcare costs in developing countries were covered through out-of-pocket (OOP) spending at the time and place of treatment. The World Health Organization and the World Bank have long advocated for lower OOP expenses and UHC. The 2030 Sustainable Development Goals (SDGs) emphasize ensuring that everyone should have access to high-quality health care without suffering economic difficulties. Despite all this movement, progress needs to be accelerated. On current trends, UHC will not be achieved by 2030 [4]. Low- and middle-income countries (LMICs) share this inequitable and inefficient health-financing status. LMICs face difficulties achieving UHC because their governments cannot support their health systems sufficiently, and OOP healthcare costs have become the most common form of healthcare funding. Public contributory health insurance is becoming increasingly popular in LMICs to lower financial obstacles to accessing and offering financial risk protection to the people [3, 5, 6]. UHC remains a fundamental challenge for African and Sub-Saharan African countries (SSA), with millions of households struggling with a high percentage of OOP in total household expenditure for health services [7–9].

CBHI schemes have been adopted in African countries, including Ethiopia and developed countries, as the first steps toward achieving national health insurance coverage. It has become one of the most important risk-mitigation programs and is expected to play a significant role in assisting the country’s transition to universal health care [1, 10]. In several parts of Sub-Saharan Africa, the CBHI scheme showed some promise in improving access to healthcare for the predominantly disadvantaged rural populations and informal sector [5, 11, 12]. However, population enrollment into CBHI and membership renewals remain stubbornly low in SSA [13]. The CBHI scheme in Ethiopia was formed to improve access to health care and lower out-of-care quality [14, 15]. However, health-seeking behavior and access to modern health care are low [16].

Ethiopia has made rapid economic progress in recent decades, but it remains a developing country with a high disease burden. Ethiopia’s health system is challenged by a lack of funding and relies heavily on international aid. Due to a lack of resources in the healthcare system, healthcare services are underutilized. Compared to other low-income African countries, the Ethiopian government’s health spending is quite low [17, 18]. To alleviate the low level of health care service utilization and improve access to quality health services in an equitable, efficient, and sustainable way, the government of Ethiopia launched CBHI schemes for the agriculture and informal sector in 2011 [19]. For the first time, the program was implemented in 13 rural districts across the country’s four main regions (Tigray, Amhara, Oromia, and SNNPR) [20, 21]. It was implemented to decrease financial stress resulting from unexpected OOP payments. In Ethiopia, OOP spending accounts for a significant proportion of health sector spending. In 2013, 90.6% of private health expenditure in Ethiopia was from OOP. Despite the government’s efforts, the CBHI healthcare services utilization rate still failed to achieve the expected goal [22]. Ethiopian Demographic and Health Survey (EDHS) 2016 shows that health insurance coverage is extremely low [23].

Even though Ethiopia has been implementing the CBHI scheme since 2011 to promote the health of poor rural and urban informal residents, the enrollment rate is still very low and is affected by multiple factors [15, 24]. Therefore, further studies on CBHI enrollment were needed. There was more research on CBHI utilization in Ethiopia. However, these findings were local area studies (not done at the country level). Using a country representative sample has several advantages over other local area studies in terms of identifying the total magnitude to display national level characteristics, which could contribute to policy decisions at the national level. However, large-scale evidence supporting this fact is lacking in Ethiopia.

Moreover, previous local and national studies conducted in the region addressed specific types of insurance on a small scale using a small sample size and binary logistic regression analysis, which did not consider the random-effect/variation (the variation in CBHI enrollment between communities). Furthermore, no published literature is addressed the coverage of CBHI at the country level. Thus, to fill the existing gap, the current study aimed to assess the individual and community level factors associated with community-based health insurance enrollment in Ethiopia using multi-level analysis.

## Materials and methods

### Study design, setting, and period

Ethiopia is Africa’s second-most populous country. Ethiopia has nine administrative regions (Tigray, Afar, Amhara, Oromia, Somalia, Benishangul-Gumuz, SNNPR, Gambella, and Harari) and two administrative cities (Addis Ababa and Diredawa). Ethiopia shares borders with Eritrea in the north, Kenya and Somalia in the south, South Sudan and North Sudan in the west, and Djibouti and Somalia in the east. A community-based cross-sectional study was conducted in Ethiopia from March 2019 to June 2019. The source of data for the study was Ethiopian Mini Demographic Health Survey (EMDHS) data. EMDHS is the country representative sample survey carried out between EDHS. In two steps, the probability proportional stratified sampling method was used to choose the sample for the 2019 Ethiopian mini-DHS. In the first stage, the nine regions of Ethiopia were divided into urban and rural areas, resulting in 21 sampling strata. In all nine regions of Ethiopia, 305 enumeration areas have completed the first stage (93 are urban and the remaining 212 are rural). The household listing was conducted from January to April 2019. In the second stage, 30 fixed numbers of households within each stratum were chosen using probability proportional stratified sampling. And the enumeration areas were selected to have an equal chance of being selected from the household listings done in January-April 2019. For the sample, 9,150 houses were chosen, of which 8,794 were occupied. 8,663 out of the occupied homes were successfully reached for interviews, yielding a response rate of 99%. We downloaded secondary data from the DHS website (www.dhsprogram.com) after permission was granted through an online request to explain the objective of our study. The detailed sampling procedure has been presented in the full EMDHS 2019 report [25].

### Source and study population

The source population of this study was all Ethiopian households. During the survey year, the study population consisted of households in the country’s randomly selected enumeration areas.

### Study variables

The outcome variable of the study was community-based health insurance enrollment. It was retrieved from dichotomized EMDHS question asking for ‘enrolled in CBHI. The responses were originally classified as No (0) or yes (1).

### Independent variables

Independent predictors have classified individual and community-level factors. Individual-level factors were the sex of the head of household (Male, Female), having a mobile telephone (No, Yes), having land for Agriculture (No, Yes), owning livestock, herds, or farm animals (No, Yes), Has radio (No, Yes), Wealth index (Poorest, Poorer, Middle, Richer, Richest), Receiving cash of food from the safety Net Program (No, Yes), Education level of household head (No Education, Primary, Secondary and above), age of household heads (15–34 ages,35–54 ages,55–74 ages, > = 75 ages), Number of household members (1–3 members, 4–6 members, 7–9 members,> = 10 members), Number of children 5 and under (No child, 1–2 children, > = 3 children) and community-level factors were residence(urban, rural), and region.

### Data management and statistical analysis

The data was cleaned by STATA version 13 software. In this study, since the EMDHS data was the hierarchical structure(households (level 1) were nested within the community (level 2)) and the outcome variable had binary response categorized as Yes or No, we used a multi-level logistic regression analysis technique using STATA (version 13) software. The effect of each independent variable (both individual and community-level) on the dependent variable was checked at a p-value of 0.25. Variables with a p-value of less than 0.25 in the bivariable multilevel logistic regression analysis were considered candidates for multivariable multilevel logistic regression analysis. Adjusted Odds Ratio (AOR) with 95% CI was used to identify associated factors with the outcome variable. Those independent variables with a P-value of less than 0.05 in the multivariable logistic regression analysis were considered significantly associated with the outcome variable. In addition, the descriptive statistics were displayed for the community and individual level explanatory variables by using frequencies and percentages.

Four models were displayed in this analysis, Model I (Empty model) was fitted without explanatory variables to test random variability in the intercept and to estimate the intraclass correlation coefficient (ICC). Model II examined the effects of individual-level characteristics, model III examined the effect of community-level variables, and Model IV examined the effects of both individual and community-level characteristics simultaneously. The random effect measures variation of CBHI enrollment of households across clusters expressed by Intraclass Correlation (ICC), quantifying the degree of heterogeneity of CBHI enrollment of households between clusters. ICC was calculated as the proportion between cluster variation and total variation. The variability in the odds of CBHI enrollment explained by successive models was calculated by Proportional Change in Variance (PCV). PCV was the variability in the odds of enrolling in CBHI explained by successive models.

### Ethical consideration

Since the study was a secondary data analysis of publicly available survey data from the measure DHS program, ethical approval and participant consent were not necessary for this particular study. We requested DHS Program, and permission was granted to download and use the data for this study from (www.dhsprogram.com). We confirm that all methods followed the relevant guidelines and regulations.

## Results and discussion

### Socio-demographic and economic characteristics

A total of 8663 study participants (households) were involved. Of this, 6291 (72.6%) of the respondents were males. Most participants (68.8%) had a mobile phone and no radio (71.2%). More than half of the households (60.2%) owned livestock, herds, or farm animals. Almost half of the households had land for Agriculture (51.4%). (47.7%) of the household, heads were not educated. 31.3% of the households had a primary education status, and the remaining 21% of the respondents had secondary and above education status. 30.1% of households had the richest wealth index. 24.2% of the households had the poorest wealth index. Most households (83.1%) were not receiving cash or food from the safety net program. (42.3%) of households had four up to six household members. Half of the households (50.1%) had no children of five or under. Out of total households, 11.8%, 11.7%, 11.6%, and 7.6% were from Oromia, SNNP, Amhara, and Somali regions, respectively. The majority (69.5%) of the households were rural residents (See Tables 1 and 2). The prevalence of CBHI scheme enrollment in this study was 20.15%.

**Table 1:** Characteristics of individual-level factors of CBHI enrollment, Mini-EDHS 2019.

Individual-level characteristics	Enroll in CBHI		Total (%)
	Yes (%)	No (%)	
Sex of head of household			
Male	1352(21.5%)	4939(78.5%)	6291(72.6%)
Female	394(16.6%)	1978(83.4%)	2372(27.4%)
Has mobile telephone			
No	550(20.3%)	2154(79.7%)	2704(31.2%)
Yes	1196(20.1%)	4763(79.9%)	5959(68.8%)
Has land for Agriculture			
No	489(11.6%)	3719(88.4%)	4208(48.6%)
Yes	1257(28.2%)	3198(71.8%)	4455(51.4%)
Owns livestock, herds, or farm animals			
No	415(12.0%)	3037(88.0%)	3452(29.8%)
Yes	1331(25.5%)	3880(74.5%)	5211(60.2%)
Has radio			
No	1259(20.4%)	4911(79.6%)	6170(71.2%)
Yes	487(19.5%)	2006(80.5%)	2493(28.8%)
Wealth index			
Poorest	256(12.2%)	1837(87.8%)	2093(24.2%)
Poorer	336(23.9%)	1069(76.1%)	1405(16.2%)
Middle	435(33.9%)	850(66.1%)	1285(14.8%)
Richer	377(29.6%)	897(70.4%)	1274(14.7%)
Richest	342(13.1%)	2264(86.9%)	2606(30.1%)
Receiving cash of food from the safety Net Program			
No	1319(18.3%)	5877(81.7%)	7196(83.1%)
Yes	427(29.1%)	1040(70.9%)	1467(16.9%)
Education level of household head			
No Education	950(23.0%)	3178(77.0%)	4128(47.7%)
Primary	579(21.3%)	2136(78.7%)	2715(31.3%)
Secondary and above	217(11.9%)	1603(88.1%)	1820(21.0%)
Age of household heads			
15–34 ages	400(13.3%)	2599(86.7%)	2999(34.6%)
35–54 ages	792(22.5%)	2733(77.5%)	3525(40.7%)
55–74 ages	449(26.3%)	1257(73.7%)	1706(19.7%)
>=75 ages	105(24.2%)	328(75.8%)	433(5.0%)
Number of household members			
1–3 members	507(16.6%)	2544(83.4%)	3051(35.2%)
4–6 members	854(23.3%)	2814(76.7%)	3668(42.3%)
7–9 members	345(20.5%)	1334(79.5%)	1679(19.4%)
>=10 members	40(15.1%)	225(84.9%)	265(3.1%)
Number of children 5 and under			
No child	861(19.8%)	3479(80.2%)	4340(50.1%)
1–2 children	847(21.6%)	3077(78.4%)	3924(45.3%)
>=3 children	38(9.5%)	361(90.5%)	399(4.6%)

**Table 2:** Characteristics of community level factors of CBHI enrollment, Mini-EDHS 2019.

Community level characteristics	Enroll in CBHI		Total (%)
	Yes (%)	No (%)	
Region			
Tigray	351(49.2%)	363(50.8%)	714(8.2%)
Afar	20(3.0%)	644(97.0%)	664(7.7%)
Amhara	583(57.9%)	424(42.1%)	1007(11.6%)
Oromia	212 (20.8%)	806(79.2%)	1018(11.8%)
Somali	23(3.5%)	634(96.5%)	657(7.6%)
Benishangul-Gumuz	76(10.4%)	658(89.6%)	734(8.5%)
SNNPR	214(21.0%)	803(79.0%)	1017(11.7%)
Gambela	51(7.4%)	642(92.6%)	693(8.0%)
Harari	87(12.1%)	632(87.9%)	719(8.3%)
Addis Ababa	84(12.0%)	618(88.0%)	702(8.1%)
Dire Dawa	45(6.1%)	693(93.9%)	738(8.5%)
Residence			
urban	310(11.7%)	2335(88.3%)	2645(30.5%)
Rural	1436(23.9%)	4582(76.1%)	6018(69.5%)

### Multilevel logistic regression analysis

A two-level mixed-effect logistic regression was used to analyze the effect of individual characteristics and community-level factors in determining household CBHI enrollment. The fixed effects (a measure of association) and the random intercepts for the CBHI utilization households were presented (see Table 3).

**Table 3:** Multilevel logistic regression analysis of individual and community-level factors associated with CBHI enrollment in Ethiopia.

Characteristics	Model I	Model II	Model III	Model IV
Fixed effects		AOR(95%CI)	AOR(95%CI)	AOR(95%CI)
Sex of head of household				
Male^(ref)^		1		1
Female		0.89(0.75, 1.07)		0.94(0.78, 1.12)
Has mobile telephone				
No^(ref)^		1		1
Yes		1.25 (1.04, 1.50)		1.31(1.09, 1.57)
Has land for Agriculture				
No ^(ref)^		1		1
Yes		1.60(1.30, 1.98)		1.44(1.17, 1.78)
Owns livestock, herds, or farm animals				
No ^(ref)^		1		1
Yes		1.68(1.36, 2.07)		1.65(1.33, 2.03)
Has radio				
No ^(ref)^		1		1
Yes		1.00(0.84, 1.18)		1.03(0.87, 1.21)
Wealth index				
Poorest^(ref)^		1		1
Poorer		1.31(1.01, 1.70)		1.14(0.88, 1.48)
Middle		1.83(1.38, 2.44)		1.58(1.19, 2.10)
Richer		1.99(1.46, 2.73)		1.71(1.25, 2.33)
Richest		1.87(1.27, 2.76)		1.57(1.04, 2.37)
Receiving cash of food from the safety Net Program				
No ^(ref)^		1		1
Yes		2.86(2.32, 3.54)		3.22(2.59, 3.98)
Education level of household head				
No Education^(ref)^		1		1
Primary		1.17(0.99, 1.39)		1.17(0.98, 1.39)
Secondary and above		0.98(0.76, 1.26)		1.00 (0.78, 1.29)
Age of household heads				
15–34 ages^(ref)^		1		1
35–54 ages		1.44(1.19, 1.74)		1.41(1.17, 1.71)
55–74 ages		1.69(1.34, 2.14)		1.66(1.32, 2.10)
>=75 ages		1.34(0.95, 1.89)		1.31(0.93, 1.85)
Number of household members				
1–3 members^(ref)^		1		1
4–6 members		1.23(1.03, 1.49)		1.27(1.06, 1.53)
7–9 members		1.15(0.89, 1.47)		1.23(0.96, 1.57)
>=10 members		1.23(0.76, 1.98)		1.36(0.84, 2.19)
Number of children 5 and under				
No child^(ref)^		1		1
1–2 children		1.12(0.95, 1.33)		1.11(0.94, 1.32)
>=3 children		0.61(0.39, 0.95)		1.65(1.42, 2.03)
Region				
Tigray^(ref)^			1	1
Afar			0.02(0.01, 0.04)	0.02(0.01, 0.04)
Amhara			1.49(0.75, 2.97)	1.64(0.85, 3.19)
Oromia			0.19(0.09, 0.39)	0.16(0.08, 0.32)
Somali			0.02(0.01, 0.05)	0.02(0.01, 0.06)
Benishangul-Gumuz			0.06(0.03, 0.14)	0.071(.03, 0.15)
SNNPR			0.17(0.08, 0.34)	0.16(0.08, 0.31)
Gambela			0.05(0.02, 0.11)	0.06(0.03, 0.13)
Harari			0.10(0.05, 0.22)	0.09(0.04, 0.20)
Addis Ababa			0.11(0.05, 0.26)	0.13(0.05, 0.29)
Dire Dawa			0.05(0.02, 0.11)	0.05(0.02, 0.10)
Residence				
urban ^(ref)^			1	1
Rural			1.35(0.86, 2.11)	0.85(0.52, 1.39)
Random effects	Model I	Model II	Model III	Model IV
Community variance (SE)	3.95	3.51	1.59	1.43
ICC (%)	54.57	51.59	32.64	30.25
PCV (%)	NA	11.14	59.75	63.79
Model fitness				
Log likelihood	-3211.85	-3063.99	-3113.99	-2965.79
AIC	6427.69	6171.98	6253.99	5997.58
BIC	6441.83	6327.45	6345.86	6230.79

Note: AOR = Adjusted odds ratio; AIC = Akaike Information Criteria; BIC = BIC; Bayesian Information Criteria; CI = Confidence Interval; ICC = Intra Class Correlation; PCV = Proportional Change in Variance; Ref = reference categories.

As depicted in the empty model, 54.57% of the total variance in the odds of CBHI enrollment was accounted for between cluster variations of characteristics. The between cluster variability declined over successive models from 54.57% in the empty model to 51.59% in an individual-level only model, 32.64% in the community-level factors only model, and 30.25% in the combined model. Thus, the combined model of individual-level and community-level factors were preferred for enrolling in the community-based health insurance program. In model II, only individual-level variables were added. The result showed the age of household heads, Number of children 5 and under, Number of household members, having land for Agriculture, having a mobile telephone, Receiving cash of food from the safety Net Program, owning livestock, herds of farm animals, Wealth index Region were significantly affected the community-based health insurance enrollment of households. The ICC in Model II indicated that 51.59% of the variation in CBHI enrollment was attributable to differences across the communities. As shown by the PCV, 11.14% of the variance in CBHI enrollment across communities was explained by the individual-level characteristics. In Model III, only community-level variables (residence and region) were added. The ICC in Model III implied that differences between communities account for about 32.64% of the variation in CBHI enrollment. In addition, the PCV indicated that community-level characteristics explained 59.75% of the variation in CBHI enrollment between communities. Model IV, the final model, simultaneously included individual and community level characteristics. As shown by the estimated ICC, 30.25% of the variability in CBHI enrollment was attributable to differences between communities. The PCV indicated that individual and community-level factors explained 63.79% of the variation in the CBHI enrollment across communities.

After controlling for other individual and community level factors, households with land for Agriculture were 1.44 times (AOR = 1.44; 95%CI 1.17, 1.78) more likely to enroll in the CBHI scheme than households who had no land for Agriculture. Household heads with mobile telephones 1.31 times (AOR = 1.31; 95% CI 1.09, 1.57) were more likely to enroll in the CBHI scheme than household heads with no mobile telephone. Households that own livestock, herds, or farm animals were 1.65 times (AOR = 1.65; 95% CI 1.33, 2.03) more likely to enroll in the CBHI scheme than households who have not had livestock, herds, or farm animals. Regarding the age of household heads, household heads within the 35–54 and 55–74 age groups were 1.41 times (AOR = 1.41;95%CI 1.17, 1.71) more likely to enroll in the CBHI scheme and 1.66 times(AOR = 1.66;95%CI 1.32, 2.10) more likely to enroll in CBHI scheme as compared to households in the age group 15–34 ages respectively, while keeping other variables constant. After holding other factors constant, poorer, middle, richer, and richest households were 1.58 times (AOR = 1.58; 95%CI 1.19, 2.10), 1.71 times (AOR = 1.71; 95%CI 1.25, 2.33), 1.57 times (AOR;1.57; 95% 1.04, 2.37) more likely to enroll in CBHI scheme as compared to poorest households respectively. Households who received cash of food from the safety net program were 3.22 times (AOR = 3.22;95% CI 2.59, 3.98) more likely to enroll in (utilize) the CHHI scheme as compared to households who were not received cash of food from the safety net program, holding other households constant. Households with 4 up to 6 members were 1.27 times (AOR = 1.27; 95 CI% 1.06, 1.53) more likely to enroll in the CBHI scheme than households with 1 up to 3 household members while keeping other variables constant.

The number of living children 5 years old and under affects households’ CBHI enrollment. Households with more than three children were 1.65 times (AOR = 1.65; 95%CI 1.42, 2.03) more likely to enroll in the CBHI scheme than households with no child. Keeping other variables constant, households from the Afar region, Oromia region, Somali region, Benishangul-Gumuz region, SNNPR region, Gambela region, Harari region, Addis Ababa city administration, Dire Dawa city administration were less likely to enroll in CBHI programs as competed to households from Tigray region. There was no significant CBHI program enrollment difference between Amhara and Tigray regions.

## Discussion

This study was based on the data of the 2019 Mini Demographic and Health Survey conducted in Ethiopia. This study aimed to identify factors associated with CBHI enrollment in households in Ethiopia. The highest CBHI enrollment rate was reported in Amhara and Tigray regions.

The finding of this study revealed that household head age is a significant predictor of CBHI enrollment rate. Household heads aged 35–54 and 55–74 were more likely to have a CBHI rate than younger household heads. This study was in line with the previous study [18, 26, 27], which indicates the age of household age significantly positively affected CBHI enrollment. This study contradicted the previous study [28], age is not a significant predictor of CBHI scheme enrollment. This may be due to the fact that older people are more afraid of impending illness than younger people. Therefore, they get health insurance at a low cost to ensure safe health care utilization.

This study revealed that the wealth index was another factor influencing households’ CBHI enrollment, it positively affected CBHI enrollment for households. The odds of the richest households enrolled in the CBHI scheme were high. The odds of CBHI enrollment were higher among households in the richest wealth quintile. This finding is consistent with those from other settings elsewhere [29–31], which shows the richest households were more likely to use the CBHI program when compared the poorest households. In addition, it is in line with the previous studies [26, 28, 32, 33], which show income significantly positively affected CBHI enrollment. This study was not in line with the previous studies [34], poor wealth status households were more likely willing to pay/enroll for CBHI than rich household heads. In addition, this study contradicts the previous studies [18] household wealth status was not significantly correlated with CBHI enrollment. The justification for this discrepancy might be due to differences in socioeconomic status and health insurance experiences. This might be due to richest people having no limitation in terms of money to enroll in CBHI scheme.

Households who received cash of food from the safety net program had a significant positive effect on CBHI scheme enrollment. This study was in line with [35–37], which shows that participating in the safety net program increases the probability of CBHI uptake. The justification might be that the poor households prefer to join CBHI because they could not afford the expensive out-of-pocket payment or the CBHI premium was waived due to their poverty status.

This study also revealed that households with 4 up to 6 members were more likely to enroll in the CBHI scheme than households with 1 up to 3. This study was consistent with the previous studies [28, 38], which show family size had a significant positive effect on dropout of households from the CBHI scheme This study contradicts the previous study [26, 32, 39], which shows family size had a significant negative effect on CBHI enrollment. Moreover, this study was not in line with the study [31], which shows the number of family sizes in the household had no significant effect on CBHI enrollment. It may be due to households with a big family size have a higher chance of being ill and are more likely to face financial difficulties. As a result, the larger the family size, the more likely at least one member of the household would become ill, and the greater the possibility of enrolling in health insurance.

Households with more than three children five and under years old significantly positively affected CBHI scheme enrollment. This study was consistent with the previous studies [18], the number of children has had a positive effect on CBHI enrollment. This study was contradicted by [31], which shows the number of children in the household had no significant effect on CBHI enrollment. The fact that infectious diseases like malaria and diarrhea were a major cause of morbidity and mortality among children could justify this. As a result, households with fewer than five children were registered in the CBHI plan to reduce out-of-pocket expenses.

This study’s findings showed households with land for agriculture had a significant positive effect on CBHI enrollment. This study was in line with the other study [26], which shows households with the maximum amount of farming were willing to enroll in CBHI schemes. Moreover, this study was in line with the previous study [40], which shows agricultural production was positively related to the CBHI scheme. This study contradicted the study [41], which shows the size of agriculture had no significant effect on CBHI uptake. The justification for this discrepancy might be due to differences in socioeconomic status and health insurance experiences. This may be explained by the fact that the availability of agricultural land may provide some form of daily revenue, allowing them to pay insurance premiums easily.

Household heads who had mobile telephones significantly positively affected CBHI scheme enrollment. We know that a person who can read and write can usually use a mobile telephone. This result may be related to the education status of household heads. Implies that household heads who can read and write were more likely to enroll in the CBHI scheme as compared to household heads who are not able to read and write. This is in line with the study [8, 42], which shows the education of the household head was positively correlated with enrollment in CBHI. This might be due to the fact that people who could not read and write and had no good understanding of the importance of the scheme, and may not enroll in the CBHI program.

Households who own livestock, herds, or farm animals significantly positively affected CBHI scheme enrollment. This study was in line with the previous study [40, 43], which shows a significant association between owned livestock and CBHI utilization. This may be justified by the presence of cattle, herds, or farm animals that may provide some form of daily revenue, allowing them to pay insurance payments easily.

This study found geographical regions to be an important predictor of CBHI enrollment. Households from the Afar region, Oromia region, Somali region, Benishangul-Gumuz region, SNNPR region, Gambela region, Harari region, Addis Ababa city administration, Dire Dawa city administration had lower odds of CBHI scheme enrollment compared to those households residing in Tigray regions. This study was in line with previous studies [31, 44], geographical location was the key predictor of health insurance enrollment. This disparity in enrollment rate between regions could be due to socio-cultural, socio-economic, and health-care-quality issues, among other reasons.

### Limitations of the study

Potentially important factors like marital status, occupation, presence of chronically ill persons in the household, and presence of elders in the household were not accessible because the EMDHS data were secondary. In addition, Since the EMDHS was a questionnaire-based survey that depended on the respondents’ memories, recall bias could be another weakness of this study.

## Conclusion

The study found that household enrollment in community-based health insurance varied nationwide. CBHI enrollment rates were highest in Amhara and Tigray. For Afar, Oromia, Somalia, Benishangul-Gumuz, SNNPR, Gambela, Harari, Addis Ababa city administration, and Dire Dawa city administration, the CBHI membership rate was low. Individual and community-level variables were found to affect household CBHI enrollment significantly. At the individual level; Age of household heads, Number of children 5 and under, Number of household members, having land for Agriculture, having a mobile telephone, Receiving cash of food from the safety Net Program, Owning livestock, herds of farm animals, Wealth index, and at the community level; the region had a significant association with community-based health insurance enrollment. The random effects showed that the variation in community-based health insurance enrollment between communities was statistically significant. Further research will be needed to understand better why these factors may affect the enrollment of households in the scheme. In addition, since more information is needed to explore the reason for the low uptake of CBHI, further study is recommended with the application of both quantitative and qualitative study designs. Furthermore, the ministry of health, health bureaus, and other concerning bodies prioritize clusters with low health insurance coverage to strengthen health system financing and intervene in factors that negatively affect CBHI enrollment in households.

## Supporting information

S1 Data (SAV)

## Abbreviations

AIC: Akaki Information Criteria
AOR: Adjusted Odds Ratio
BIC: Bayesian Information Criteria
CBHI: Community Based Health Insurance
CI: Confidence Interval
Crude: Odds Ratio
EMDHS: Ethiopia Mini Demographic and Health Survey
ICC: Intra Class Correlation Coefficient
OR: Odds Ratio
PVC: Proportional Change in Variance
SNNPR: Southern Nations, Nationalities, and Peoples’ Region
UHC: Universal Health Coverage
WHO: World Health Organization
